# Racial differences in mantle cell lymphoma in the United States

**DOI:** 10.1186/1471-2407-14-764

**Published:** 2014-10-15

**Authors:** Yu Wang, Shuangge Ma

**Affiliations:** School of Statistics, Renmin University of China, 59 Zhongguancun Avenue, Beijing, 100872 China; School of Public Health, Yale University, 60 College ST, New Haven, CT 06520 USA

**Keywords:** Mantle cell lymphoma, Racial differences, SEER, Non-hodgkin lymphoma

## Abstract

**Background:**

MCL (mantle cell lymphoma) is a rare subtype of NHL (non-Hodgkin lymphoma) with mostly poor prognosis. Different races have different etiology, presentation, and progression patterns.

**Methods:**

Data were analyzed on MCL patients in the United States reported to the SEER (Surveillance, Epidemiology, and End Results) database between 1992 and 2009. SEER contains the most comprehensive population-based cancer information in the U.S., covering approximately 28% of the population. Racial groups analyzed included non-Hispanic whites, Hispanic whites, blacks, and Asians/PIs (Pacific Islanders). Patient characteristics, age-adjusted incidence rate, and survival rate were compared across races. Stratification by age, gender, and stage at diagnosis was considered. Multivariate analysis was conducted on survival.

**Results:**

In the analysis of patients’ characteristics, distributions of gender, marital status, age at diagnosis, stage, and extranodal involvement were significantly different across races. For all three age groups and both male and female, non-Hispanic whites have the highest incidence rates. In the analysis of survival, for cancers diagnosed in the period of 1992–2004, no significant racial difference is observed. For cancers diagnosed in the period of 1999–2004, significant racial differences exist for the 40–64 age group and stage III and IV cancers.

**Conclusions:**

Racial differences exist among MCL patients in the U.S. in terms of patients’ characteristics, incidence, and survival. More extended data collection and analysis are needed to more comprehensively describe and understand the racial differences.

**Electronic supplementary material:**

The online version of this article (doi:10.1186/1471-2407-14-764) contains supplementary material, which is available to authorized users.

## Background

NHL (non-Hodgkin lymphoma) has over 30 subtypes, with different subtypes having different clinical and molecular features [1]. MCL (mantle cell lymphoma) is a rare subtype of NHL. It was proposed as a distinct entity in 1992 [2] and accepted by the Revised European American Classification for Lymphoma (REAL) in 1994. The symptoms of MCL include swelling, loss of appetite and fatigue, night sweats, fevers, and weight loss. Diagnosis of MCL usually requires removing an enlarged lymph node and examining the cells under a microscope. MCL comprises about 6% of all NHL cases [3]. Its age-adjusted incidence rate is about 0.51 to 0.55 per 100,000 person-years. In the U.S., the incidence of MCL had been increasing between 1992 and 2007 [4, 5], and there are currently about 15,000 MCL patients in total. These patients are typically Caucasian, male, and elderly. Multiple risk factors have been suggested as associated with the risk of developing MCL, including lifestyle and occupational risk factors, viruses, family history, and molecular risk factors [6]. For MCL patients, the standard first-line treatment consists of rituximab (a chimeric monoclonal anti-CD20 antibody, approved by U.S. FDA in 1997) in combination with chemotherapy [7]. Other treatment options include other monoclonal antibody therapy, stem cell transplants, radiotherapy, steroid therapy, and relatively newer drugs such as Temsirolimus, Lenalidomide, and Bortezomib. Most MCLs have an aggressive clinical course with a median survival of 3–7 years [8]. Multiple factors influence MCL prognosis. Shorter overall survival has been associated with older age, worse ECOG (Eastern Cooperative Oncology Group) performance status, higher LDH (Lactate dehydrogenase), higher white blood cell count [9], and advanced diseases [10]. Multiple genetic factors have also been implicated in MCL prognosis.

The goal of this study, different from that of many published ones, is to comprehensively investigate the racial differences among MCL patients in the U.S., in terms of patients’ characteristics, clinical-pathologic features, incidence, and survival rates. Racial differences in multiple clinical and epidemiologic aspects for some other cancer types and other NHL subtypes, such as DLBCL (diffuse large B-cell lymphoma) and follicular lymphoma, have been studied [11–18]. However, research on MCL remains scarce. The existing MCL studies have limitations by focusing on specific racial groups or specific outcomes. For example, Chim and others [19] compared only Chinese and Caucasians; and Zhou and others [4] focused on incidence rates. This article targets filling the knowledge gap by comprehensively comparing non-Hispanic whites, Hispanic whites, blacks, and Asians/PIs (Pacific Islanders) in multiple aspects of MCL.

## Methods

### Source population

The population-based sample was obtained from the SEER (Surveillance, Epidemiology, and End Results, http://seer.cancer.gov/) database. All data analyzed in this study are publicly available. SEER is the most comprehensive population-based cancer database in the U.S., containing data from 18 regional and state registries. It has multiple registry groupings for analyses. Different registry groupings cover different numbers of regions and time periods. More details are available at http://seer.cancer.gov/registries/terms.html. SEER 9, 13, and 18 registries, which are analyzed in this article, cover approximately 9.5%, 14%, and 28.0% of the U.S. population, respectively. For each MCL case, the first matching record was selected for analysis. The International Classification of Diseases for Oncology (ICD-O)-3 code used for MCL was 9673. In data examination, we found 16 patients with T-cell MCL and 5 patients with null-cell MCL. Such samples were removed from analysis. All SEER registries were included in the analysis.

Different SEER registry groupings were used to maximize sample size. Specifically, for the analysis of patients’ characteristics and clinical-pathologic features, SEER 9 contains data on cancers diagnosed between 1973 and 2009. For incidence, SEER 13 data, which include detailed race and incidence information for cancers diagnosed between 1992 and 2009, are analyzed. And for survival, SEER 18 has data for cancers diagnosed between 1973 and 2004 and followed up to 12/31/2009. As the current definition of MCL was not established until 1992, we limited our analysis to all cancers diagnosed after 1992. Rituximab was introduced in 1997, is now the standard first-line treatment, and has a significant effect on survival. Thus, we also conducted survival analysis on cancers diagnosed after 1999 [20]. Clinical-pathologic features analyzed include gender, marital status (single, married, and separated/divorced/widowed), age at diagnosis, stage (I, II, III, and IV, according to the Ann Arbor staging system), B symptoms (no, yes, and unknown), extranodal involvement (no and yes), and survival time. The main outcomes of interest are incidence rate and survival rate.

### Statistical analysis

In the comparison of patient characteristics and clinical-pathologic features across racial groups, Chi-squared tests and ANOVA were used, and p-values were computed. The analysis was conducted using SAS version 9.2. Age-adjusted incidence rates were calculated with SEER*Stat using U.S. 2000 Census data for age-standardization. Five-year relative survival rates were calculated with SEER*Stat using an actuarial method, which accommodates the right-censored nature of survival data [21]. Multivariate Cox regressions were then conducted, adjusting for age at diagnosis, gender, marital status, B symptoms, and extranodal involvement, and stratified by stage at diagnosis.

## Results

### Patients’ characteristics and clinical-pathologic features

A total of 2,958 MCL patients were identified in SEER between 1992 and 2009 (Table 1). There are overall more male patients (67.2%). The gender distributions are different across races (p-value = 0.043), with Hispanic whites having the most male patients (70.6%) and blacks having the least (56.2%). Most MCL patients are married (68.4%). Different racial groups have significantly different marital status (p-value < 0.001). Among Asian/PI patients, 74.8% are married. In contrast, only 42.9% of black patients are married. The age at diagnosis is also significantly different across races (p-value < 0.001). For non-Hispanic whites, the mean age at diagnosis is 68.2, compared to 62.8 for blacks. Most MCLs are diagnosed at late stages. In our analysis, 62.3% are stage IV, and 14.7% are stage III. The racial difference is significant (p-value-0.029), with for example 63.2% of non-Hispanic whites and 53.5% blacks having stage IV. The distribution of B symptoms shows no racial difference. Overall, 16.2% have extranodal involvement, and the racial difference is significant (p-value < 0.001). Asians/PIs have the highest percentage of extranodal involvement. Most patients did not receive surgery or radiation (59.8%). There is no significant racial difference. The median survival time is 46.0 months. Hispanic whites have the longest median survival (57.0 months), while blacks having the shortest (39.0 months). However, the racial difference is not significant.

**Table 1:** **MCL patients’ characteristics and clinical-pathologic features for the whole cohort and different racial groups**

	Total (n = 2958)	Non-Hispanic white (n = 2620)	Hispanic white (n = 109)	Black (n = 121)	Asian/PI (n = 108)	***P***
**Gender**						0.043
Male	1989 (67.2)	1775 (67.7)	77 (70.6)	68 (56.2)	69 (63.9)	
Female	969 (32.8)	845 (32.3)	32 (29.4)	53 (43.8)	39 (36.1)	
**Marital Status**						<0.001
Single	243 (8.7)	195 (7.9)	20 (20.8)	21 (18.8)	7 (6.8)	
Married	1905 (68.4)	1721 (69.6)	59 (61.5)	48 (42.9)	77 (74.8)	
Separated/divorced/widowed	636 (22.8)	557 (22.5)	17 (17.7)	43 (38.4)	19 (18.4)	
**Age at diagnosis**	67.8 ± 12.3	68.2 ± 12.1	64.3 ± 12.7	62.8 ± 13.7	67.0 ± 12.1	<0.001
**Stage**						0.029
Stage I	396 (14.1)	338 (13.6)	20 (20.2)	18 (15.8)	20 (18.7)	
Stage II	251 (8.9)	219 (8.8)	8 (8.1)	9 (7.9)	15 (14.0)	
Stage III	411 (14.7)	357 (14.4)	17 (17.2)	26 (22.8)	11 (10.3)	
Stage IV	1747 (62.3)	1571 (63.2)	54 (54.5)	61 (53.5)	61 (57.0)	
**B symptoms**						0.108
No	1070 (36.2)	949 (36.2)	47 (43.1)	36 (29.8)	38 (35.2)	
Yes	596 (20.1)	513 (19.6)	23 (21.1)	35 (28.9)	25 (23.1)	
Unknown	1292 (43.7)	1158 (44.2)	39 (35.8)	50 (41.3)	45 (41.7)	
**Extranodal involvement**						<0.001
No	2478 (83.8)	2206 (84.2)	93 (85.3)	106 (87.6)	73 (67.6)	
Yes	480 (16.2)	414 (15.8)	16 (14.7)	15 (12.4)	35 (32.4)	
**Survival time** (month) (Median ± SD)	46.0 ± 1.8	46.0 ± 1.9	57.0 ± 13.5	39.0 ± 7.9	46.0 ± 6.8	0.735

Cancers diagnosed between 1992 and 2009 in the SEER 9 database. For a continuous variable, mean ± standard deviation, and for a categorical variable, count (percentage).

### Incidence

The overall age-adjusted incidence rate is 0.64 per 100,000 person-years (Table 2). Incidence increases with age. The three age groups have incidence rates 0.01, 0.73, and 3.22, respectively. Overall, non-Hispanic whites have the highest incidence rate (0.73, 95% CI 0.71-0.76), significantly higher than those of the other races. For the <40 age group, the incidence rates are low for all races. For the 40–64 and 65+ age groups, non-Hispanic whites have the highest incidence rates, followed by Hispanic whites. Asians/PIs have the lowest rates. Overall, males have a higher incidence rate (0.98, 95% CI 0.94-1.02) than females (0.37, 95% 0.35-0.39). For both male and female, non-Hispanic whites have the highest incidence rates, followed by Hispanic whites and then blacks.

**Table 2:** **Age-adjusted MCL incidence rates per 100,000 person-years for the whole cohort and different racial groups, stratified by age and gender**

Incidence	Non-Hispanic white		Hispanic white		Black		Asian/ PI		Total	
	n	IR (95% CI)	n	IR (95% CI)	n	IR (95% CI)	n	IR (95% CI)	n	IR (95% CI)
All ages	3320	0.73 (0.71-0.76)	328	0.53 (0.48-0.60)	180	0.32 (0.28-0.38)	198	0.29 (0.26-0.34)	4073	0.64 (0.62-0.66)
<40 years	35	0.02 (0.01-0.02)	11	0.01 (0.01-0.02)	5	0.01 (0.00-0.02)	1	0.00 (0.00-0.01)	53	0.01 (0.01-0.02)
40-64 years	1195	0.87 (0.82-0.92)	138	0.57 (0.47-0.67)	87	0.43 (0.34-0.53)	78	0.34 (0.27-0.42)	1517	0.73 (0.69-0.77)
65+ years	2090	3.66 (3.50-3.82)	179	2.80 (2.40-3.24)	88	1.49 (1.19-1.83)	119	1.51 (1.25-1.80)	2503	3.22 (3.09-3.35)
Male	2243	1.13 (1.08-1.17)	234	0.85 (0.74-0.98)	108	0.45 (0.37-0.55)	131	0.44 (0.37-0.52)	2745	0.98 (0.94-1.02)
Female	1077	0.43 (0.40-0.45)	94	0.29 (0.23-0.35)	72	0.23 (0.18-0.29)	67	0.18 (0.14-0.23)	1328	0.37 (0.35-0.39)

Diagnoses in the period of 1992–2009 in the SEER 13 database. In each cell, estimate (95% CI). Rates were age-adjusted using the U.S. 2000 Census population.

### Survival

**Figure 1:**
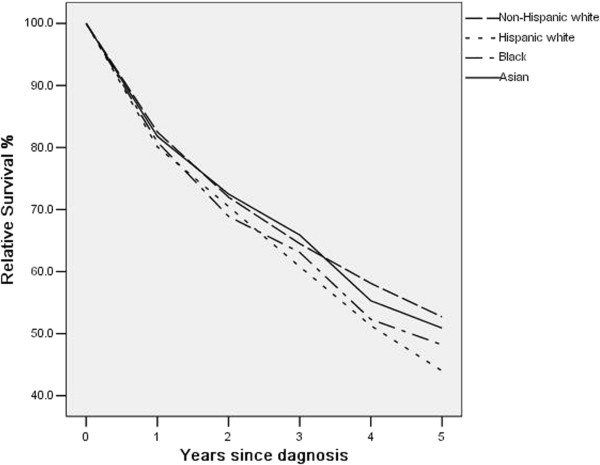
**Relative survival up to five years for different racial groups.** Cancers diagnosed in the period of 1992–2004 and followed up to 12/31/2009.

For cancers diagnosed in the period of 1992–2004, the five-year relative survival rates are shown in Table 3. When stratified by age, for the <40 years group, Asians/PIs have the best five-year survival rate (100%), whereas blacks having the worst (45.5%). For the 40–64 years group, non-Hispanic whites have the best survival rate (63.1%), while Hispanic whites have the worst (51.4%). For the 65+ years group, non-Hispanic whites have the best survival rate (44.4%), while blacks have the worst (34.9%). The racial differences are not significant in the multivariate analysis (detailed results are shown in Additional file 1: Table S4). When stratified by gender, for male, non-Hispanic whites have the best survival rate, and Hispanic whites have the worst. For female, Asians/PIs have the best survival rate, and Hispanic whites still have the worst. The racial differences are not significant in multivariate analysis. When stratified by stage at diagnosis, for stage I-III, Asians/PIs have the best survival, followed by non-Hispanic whites. Hispanic whites have the worst survival. The racial differences are not significant. Again these results should be taken cautiously because of the small sample sizes. For stage IV, the racial difference is borderline significant in multivariate analysis (p-value = 0.063). In particular, non-Hispanic whites have the best survival rate (49.0%), followed by blacks (45.4%). Figure 1 shows the unadjusted survival rate for five years. In the first year, the differences across races are ignorable. Between year one and year three, there is no dominating racial group. The survival curves are more separated between year four and year five, with Hispanic whites having the worst and non-Hispanic whites having the best survival.

**Table 3:** **Five-year relative survival rates for different racial groups, stratified by age, gender, and stage at diagnosis**

	Total (n = 5438)		Non-Hispanic white (n = 4458)		Hispanic white (n = 446)		Black (n = 241)		Asian/PI (n = 219)		P-value
	n	Rate (95% CI)	n	Rate (95% CI)	n	Rate (95% CI)	n	Rate (95% CI)	n	Rate (95% CI)	
**Age group**											
<40 years	75	78.0 (64.8-86.7)	48	84.7 (69.6-92.7)	12	66.8 (26.5-88.5)	11	45.5 (7.8-78.4)	3	100.0	0.7
40-64 years	2257	61.9 (59.4-64.3)	1802	63.1 (60.3-65.8)	202	51.4 (42.3-59.7)	126	58.0 (46.8-67.6)	97	61.2 (48.8-71.4)	0.523
65+ years	3106	43.2 (40.7-45.7)	2608	44.4 (41.7-47.1)	232	35.5 (26.5-44.6)	104	34.9 (22.5-47.5)	119	41.2 (29.7-52.3)	0.762
**Gender**											
Male	3740	51.2 (49.0-53.3)	3062	52.2 (49.8-54.5)	330	43.6 (36.0-50.9)	152	49.5 (39.0-59.2)	148	46.6 (36.4-56.2)	0.487
Female	1698	53.1 (49.9-56.1)	1396	54.9 (51.9-57.9)	116	44.4 (32.6-55.5)	89	45.4 (31.9-58.0)	71	59.5 (44.2-71.9)	0.259
**Stage at diagnosis**											
Stage I	633	72.6 (67.2-77.2)	518	72.9 (66.9-78.0)	46	64.2 (40.6-80.3)	27	72.1 (41.6-88.5)	33	73.7 (51.2-87.0)	0.604
Stage II	447	59.0 (52.7-64.8)	365	59.7 (52.6-66.0)	36	42.1 (18.7-64.0)	17	46.0 (14.4-73.2)	24	72.2 (45.3-87.5)	0.983
Stage III	780	45.6 (40.7-50.2)	625	46.0 (40.6-51.2)	70	39.1 (24.6-53.3)	50	40.4 (23.2-57.0)	28	62.0 (37.1-79.4)	0.143
Stage IV	3243	47.9 (45.6-50.1)	2690	49.0 (46.5-51.5)	263	39.8 (31.8-47.7)	131	45.4 (34.2-55.9)	125	37.6 (26.6-48.5)	0.063

Cancers diagnosed in the period of 1992–2004 and followed up to 12/31/2009 in the SEER 18 database. In each cell, estimated rate (95% CI). P-values were generated from multivariate Cox models. Details are provided in Additional file 1.

For cancers diagnosed in the period of 1999–2004, the survival analysis results are presented in Additional file 1: Tables S5 and Table S6. When stratified by age, the racial difference is significant for the 40–64 years group (p-value = 0.025). Specifically, non-Hispanic whites have the best survival (64.4%), while Hispanic whites have the worst (50.7%). When stratified by gender, the racial difference is borderline significant for female (p-value = 0.093), with non-Hispanic whites having the best survival (54.0%) and Hispanic whites having the worst (42.2%). When stratified by stage at diagnosis, the racial differences are significant for stage III and IV. In particular, for stage III, Asians/PIs have the best survival, while Hispanic whites have the worst. For stage IV, non-Hispanic whites have the best survival (50.5%), followed by blacks (44.7%). Asians/PIs have the worst survival (38.7%).

## Discussion

### Main findings and possible interpretations

As a mostly aggressive variant of NHL, MCL has been studied in multiple publications. However, most of the existing studies have been focused on pathogenesis, incidence trends, and survival patterns. In some studies, race has been included as a confounding variable. A small number of studies are concerned with racial differences, but in a much less systematic manner. In this study, we have analyzed SEER data, comprehensively compared non-Hispanic whites, Hispanic whites, blacks, and Asians/PIs, and found that for MCL patients in the U.S., racial differences exist in terms of patients’ characteristics, clinical-pathologic features, incidence, and survival. As mentioned in Introduction, racial differences have been examined for other cancer types and other subtypes of NHL. It has been suggested in the literature [22] that MCL differs from other NHL subtypes in many aspects. Thus in this study, we have focused on MCL without attempting to compare with findings on other NHL subtypes.

The observed gender and age distributions are similar to those in the literature. Our analysis suggests that the distributions of gender, marital status, age at diagnosis, stage, and extranodal involvement are different across racial groups. The development of MCL is an extremely complex process. The observed racial differences reflect the complex interactions of genetic makeup, occupational exposures, infectious conditions, development of autoimmune diseases, family history, and socioeconomic status [23, 24]. It is unclear if there is a direct link between marital status and MCL. In the general population, different racial groups have significantly different marital status [25]. Specifically, according to the National Healthy Marriage Resource Center, the percentages of ever married are 72.6% (white men), 56.7% (black men), 66.6 (Asian men), 60.0% (Hispanic men), 79.3% (white women), 58.1% (black women), 74.7% (Asian women), and 70.3% (Hispanic women). Thus the observed significant marital status difference may or may not be relevant to MCL.

In the incidence analysis, the <40 years age group has a small sample size, and the analysis results should be interpreted with extreme caution. Other groups have moderate to large sample sizes, and the results can be more reliable. The observed incidence rate is slightly higher than that reported in the literature. Racial differences in MCL incidence have been studied in a few publications. Zhou and others [4] analyzed data on patients diagnosed between 1992 and 2004, and concluded that the incidence of MCL was higher in men than in women, and higher in Caucasians than in blacks. Aschebrook-Kilfoy and others [26] compared MCL incidence between 1992–1994 and 2005–2009, and found that the increase in incidence was strongest for men and for whites. Our analysis includes more racial groups and also provides detailed results on three age groups. Multiple factors are involved in the development of MCL and NHL overall. However, there is a lack of consensus [1, 22, 24]. Lifestyle risk factors have been suggested as associated with NHL overall, and such associations may differ across races [1]. For example, it has been suggested that certain dietary intake [27] and dietary patterns [28] have race-specific effects on the risk of NHL. A challenge is that although the aforementioned studies include MCL, because of small sample sizes, MCL-specific analysis has not been conducted. Recreational exposure to ultraviolet radiation and occupational exposure to pesticides, solvents, and gasoline also contribute to the incidence of MCL [29, 30]. It has been suggested that the racial differences in MCL is at least partly confounded by occupation [31]. The development of infectious diseases and immune suppression may also contribute to MCL risk in a race-specific way. For example, Koshiol and others [23] showed that blacks had a higher risk of NHL associated with infections than whites and a tendency toward higher risk associated with allergies. The genetic hallmark of MCL is the t(11;14) (q13;q32) translocation. Beyond this translocation, MCL tumor cells may also carry a high number of secondary chromosomal and molecular alterations [24]. For NHL overall, it has been suggested that the associations between genetic variants and risk vary across races [32]. In addition, although it is believed that most MCL cells carry the t(11;14) translocation, a study on Chinese patients showed that only 18.2% carried this translocation [33]. For other genetic variations, the racial differences remain to be explored. Another possible contributing factor of the racial differences in incidence is the racial variation in diagnosis [34].

Racial differences in survival exist for many types of cancers. For NHL overall, there has been progress in reducing disparities in survival between non-Hispanic whites and minorities [34]. MCL survival also depends on multiple factors [22]. In our analysis on cancers diagnosed in the period of 1999–2004, when stratified by age, the numbers of patients in the <40 years and 40–64 years groups are relatively small (Table 3). For the 65+ group, the unadjusted survival rates differ by as much as 9.5% (44.4% for non-Hispanic whites versus 34.9% for Hispanic whites). However, the racial difference is not significant in multivariate analysis. The lack of significant difference is also observed for both male and female. When stratified by stage at diagnosis, the racial difference is borderline significant for stage IV tumors. Such difference may be caused by multiple factors. MIPI, the MCL International Prognostic Index, contains four variables, namely age, ECOG performance status, LDH/ULN (upper limit of normal), and white blood cell counts [9]. In Table 1, we observe significant racial differences in age at diagnosis, which, according to MIPI, may contribute to the observed survival differences. In addition, white blood cell counts also vary across races, for example, blacks tend to have lower white blood cell counts than Caucasians. Other possible prognostic factors include Ki-67, MCL cell types, and Beta-2 microglobulin [35]. However such variables are not available in SEER and cannot be accounted for. As with etiology, genetic changes have also been implicated in prognosis [36, 37]. The existing genetic studies usually have small sample sizes. To the best of our knowledge, race has not been accounted for in these studies. Another possible contributing factor is treatment strategy—different racial groups can have different treatments. More racial differences are observed in the analysis of cancers diagnosed in the period of 1999–2004. In the literature, it has been suggested that the incidence and clinical-pathologic features of MCL vary over time. More importantly, the effect of rituximab and other newer treatments on MCL survival has been noted in multiple published studies.

### Limitations

The SEER database is analyzed as it is the largest cancer registry in the U.S. Even so, as can be seen from the tables, sample sizes for certain subsets are still small. In addition, using SEER has the following limitations. With multiple sites, errors may arise in tumor classification and staging. However, we do not expect a series of systematic errors correlated with ethnicity. This study may have also been hindered by the multiple coexisting classification schemes. Patients diagnosed before 2001 may have diagnosis codes from earlier ICD-O versions that need to be converted to the ICD-O-3, which may have resulted in a higher proportion of unclassified cases. Clarke and others [38] compared computer-converted ICD-O-3 codes with ICD-O-3 codes generated directly from diagnostic pathology reports, and found that the classification of MCL might have a reliability problem. Furthermore, data collected in SEER may not be comprehensive enough. Quite a few variables that are potentially associated with etiology and prognosis are not available. Information on treatment is lacking. Newer treatment regimens are not included, and there is no information on chemotherapy. In addition, insurance status, socioeconomic status, and treatment availability, which are relevant to treatment and so survival, are not measured. All patients are from the U.S. In the literature there are studies investigating the characteristics, incidence, and survival of MCL in other countries and regions [39–41]. A cross-region comparison is interesting but beyond our scope. In data analysis, we have followed published studies and simply used 0.05 as the p-value cutoff for significance. In stratified analysis, multiple comparison adjustment may be needed [42], which results in a stricter p-value cutoff.

## Conclusion

Analysis of the SEER data shows that racial differences exist among MCL patients in the U.S. in terms of patients’ characteristics, incidence, and survival. Although there are multiple possible explanations, the exact causes of the observed differences remain to be identified. More comprehensive data collection and analysis are needed to fully decipher and interpret the racial differences. Despite several limitations, findings in this study can be informative to cancer epidemiologists, clinicians, and policy-makers.

## Electronic supplementary material

Additional file 1: Table S4: Multivariate Cox regression analysis of survival, stratified by stage at diagnosis. **Table S5.** Five-year relative survival rates for cancers diagnosed in the period of 1999–2004, for different racial groups and stratified by age, gender, and stage at diagnosis. **Table S6.** Multivariate Cox regression analysis of survival for cancers diagnosed in the period of 1999–2004, stratified by stage at diagnosis. (DOCX 34 KB)
