# Supplementary material for: Racial differences in mantle cell lymphoma in the United States
Source: BMC Cancer. 2014 Oct 15;14:764. doi: 10.1186/1471-2407-14-764 (PMC4210548; doi:10.1186/1471-2407-14-764)
Supplement: Supplementary file 1 — Additional file 1: Table S4: Multivariate Cox regression analysis of survival, stratified by stage at diagnosis. Table S5. Five-year relative survival rates for cancers diagnosed in the period of 1999–2004, for different racial groups and stratified by age, gender, and stage at diagnosis. Table S6. Multivariate Cox regression analysis of survival for cancers diagnosed in the period of 1999–2004, stratified by stage at diagnosis. (DOCX 34 KB) [file 12885_2014_4945_MOESM1_ESM.docx]

**Additional file 1**

**Table S4.** Multivariate Cox regression analysis of survival, stratified by stage at diagnosis.

|  | **Stage I** | | | **Stage II** | | | **Stage III** | | | **Stage IV** | | |
| --- | --- | --- | --- | --- | --- | --- | --- | --- | --- | --- | --- | --- |
|  | **HR** | **95% CI** | ***P*** | **HR** | **95% CI** | ***P*** | **HR** | **95% CI** | ***P*** | **HR** | **95% CI** | ***P*** |
| **Age at diagnosis** | 1.053 | 1.039-1.068 | 0.000 | 1.036 | 1.019-1.054 | 0.000 | 1.045 | 1.032-1.058 | 0.000 | 1.043 | 1.037-1.049 | 0.000 |
| **Gender** |  |  |  |  |  |  |  |  |  |  |  |  |
| Male | 1 |  |  | 1 |  |  | 1 |  |  | 1 |  |  |
| Female | 0.736 | 0.539-1.005 | 0.053 | 0.809 | 0.542-1.207 | 0.299 | 0.904 | 0.677-1.206 | 0.491 | 0.763 | 0.662-0.878 | 0.000 |
| **Marital Status** |  |  |  |  |  |  |  |  |  |  |  |  |
| Single | 1 |  |  | 1 |  |  | 1 |  |  | 1 |  |  |
| Married | 0.457 | 0.275-0.760 | 0.003 | 1.237 | 0.612-2.500 | 0.553 | 0.828 | 0.495-1.384 | 0.471 | 0.874 | 0.690-1.108 | 0.266 |
| Separated/divorced/widowed | 0.707 | 0.406-1.233 | 0.222 | 2.135 | 1.008-4.523 | 0.048 | 0.947 | 0.533-1.684 | 0.853 | 1.015 | 0.780-1.321 | 0.912 |
| **Ethnic group** |  |  |  |  |  |  |  |  |  |  |  |  |
| Non-Hispanic white | 1 |  |  | 1 |  |  | 1 |  |  | 1 |  |  |
| Hispanic white | 0.846 | 0.392-1.825 | 0.670 | 0.000 | . | . | 1.612 | 0.879-2.958 | 0.123 | 1.162 | 0.772-1.747 | 0.472 |
| Black | 0.903 | 0.453-1.797 | 0.771 | 0.756 | 0.180-3.174 | 0.702 | 1.557 | 0.918-2.640 | 0.101 | 1.401 | 1.022-1.921 | 0.036 |
| Asian | 1.505 | 0.777-2.913 | 0.225 | 0.953 | 0.455-1.996 | 0.899 | 0.642 | 0.202-2.042 | 0.452 | 1.323 | 0.951-1.840 | 0.096 |
| **Treatment** |  |  |  |  |  |  |  |  |  |  |  |  |
| No surgery or radiation | 1 |  |  | 1 |  |  | 1 |  |  | 1 |  |  |
| Surgery | 0.886 | 0.642-1.223 | 0.462 | 0.843 | 0.561-1.266 | 0.410 | 0.736 | 0.553-0.979 | 0.035 | 0.858 | 0.740-0.996 | 0.044 |
| Radiation | 0.796 | 0.523-1.213 | 0.289 | 0.580 | 0.338-0.993 | 0.047 | 0.657 | 0.303-1.420 | 0.285 | 1.264 | 0.967-1.651 | 0.087 |
| Radiation & Surgery | 0.590 | 0.331-1.049 | 0.072 | 0.411 | 0.186-0.912 | 0.029 | 0.921 | 0.446-1.904 | 0.825 | 1.187 | 0.830-1.698 | 0.347 |
| **B symptoms** |  |  |  |  |  |  |  |  |  |  |  |  |
| No | 1 |  |  | 1 |  |  | 1 |  |  | 1 |  |  |
| Yes | 1.635 | 0.957-2.793 | 0.072 | 1.708 | 0.990-2.947 | 0.054 | 1.893 | 1.329-2.696 | 0.000 | 1.614 | 1.362-1.912 | 0.000 |
| Unknown | 1.227 | 0.899-1.674 | 0.198 | 1.301 | 0.883-1.917 | 0.183 | 1.334 | 0.995-1.788 | 0.054 | 1.160 | 0.996-1.351 | 0.056 |
| **Extranodal involvement** |  |  |  |  |  |  |  |  |  |  |  |  |
| No | 1 |  |  | 1 |  |  | 1 |  |  | 1 |  |  |
| Yes | 0.552 | 0.400-0.763 | 0.000 | 0.898 | 0.601-1.343 | 0.601 | 0.740 | 0.425-1.288 | 0.286 | 0.887 | 0.721-1.092 | 0.259 |

Cancers diagnosed 1992-2004, and followed up to 12/31/2009. HR: hazard ratio.

**Table S5.** Five-year relative survival rates for cancers diagnosed in the period of 1999-2004, for different racial groups and stratified by age, gender, and stage at diagnosis.

|  | **Total (n=4572)** | | **Non-Hispanic white (n=3737)** | | **Hispanic white (n=393)** | | **Black (n=193)** | | **Asian/PI (n=182)** | | **P-value** |
| --- | --- | --- | --- | --- | --- | --- | --- | --- | --- | --- | --- |
|  | n | Rate (95%CI) | n | Rate (95%CI) | n | Rate (95%CI) | n | Rate (95%CI) | n | Rate (95%CI) |  |
| **Age group** |  |  |  |  |  |  |  |  |  |  |  |
| <40 years | 49 | 90.3  (73.2- 96.7) | 32 | 92.6  (69.6- 98.4) | 7 | 83.5  (27.0- 97.5) | 7 | 85.8  (33.3-97.9) | 3 | 100.0 | 0.746 |
| 40-64 years | 1895 | 62.7  (59.8- 65.4) | 1505 | 64.4  (61.2- 67.4) | 185 | 50.7  (41.0-59.7) | 103 | 57.6  (45.0-68.3) | 77 | 57.5  (42.7-69.7) | 0.523 |
| 65+ years | 2628 | 43.6  (40.8- 46.4) | 2200 | 45.2  (42.1- 48.3) | 201 | 33.0  (23.2-43.1) | 83 | 35.6  (21.5-50.0) | 102 | 34.7  (22.4-47.3) | 0.762 |
|  |  |  |  |  |  |  |  |  |  |  |  |
| **Gender** |  |  |  |  |  |  |  |  |  |  |  |
| Male | 3203 | 52.1  (49.7-54.5) | 2612 | 53.5  (50.8-56.1) | 294 | 43.1  (34.8-51.1) | 126 | 49.4  (37.5-60.3) | 127 | 43.1  (31.7-54.0) | 0.487 |
| Female | 1369 | 52.8  (49.2-56.4) | 1125 | 54.0  (49.9-57.9) | 99 | 42.2  (29.4-54.5) | 67 | 50.1  (33.9-64.3) | 55 | 50.5  (32.1-66.4) | 0.259 |
|  |  |  |  |  |  |  |  |  |  |  |  |
| **Stage at diagnosis** |  |  |  |  |  |  |  |  |  |  |  |
| Stage I | 474 | 70.9  (64.1-76.6) | 392 | 72.9  (65.4-79.0) | 33 | 58.7  (28.8-79.6) | 21 | 68.0  (35.4-86.7) | 21 | 50.9  (23.7-72.8) | 0.604 |
| Stage II | 369 | 58.5  (51.2-65.1) | 302 | 59.9  (51.8-67.1) | 31 | 36.7  (12.5-61.7) | 15 | 56.5  (17.8-82.8) | 18 | 59.8  (28.7-80.9) | 0.983 |
| Stage III | 641 | 48.9  (43.3-54.3) | 516 | 49.8  (43.4-55.8) | 60 | 34.6  (19.6-50.1) | 36 | 48.3  (25.5-67.9) | 22 | 73.7  (44.7-89.1) | 0.143 |
| Stage IV | 2807 | 49.3  (46.7-51.8) | 2313 | 50.5  (47.7-53.2) | 241 | 41.8  (33.2-50.3) | 109 | 44.7  (32.4-56.2) | 112 | 38.7  (26.5-50.7) | 0.063 |

Cancers diagnosed in the period of 1999-2004 and followed up to 12/31/2009 in the SEER 18 database. In each cell, estimated rate (95% CI). P-values were generated from multivariate Cox models. Details are presented in Table 8.

**Table S6.** Multivariate Cox regression analysis of survival for cancers diagnosed in the period of 1999-2004, stratified by stage at diagnosis.

|  | **Stage I** | | | **Stage II** | | | **Stage III** | | | **Stage IV** | | |
| --- | --- | --- | --- | --- | --- | --- | --- | --- | --- | --- | --- | --- |
|  | **HR** | **95% CI** | ***P*** | **HR** | **95% CI** | ***P*** | **HR** | **95% CI** | ***P*** | **HR** | **95% CI** | ***P*** |
| **Age at diagnosis** | 1.049 | 1.027-1.072 | 0.000 | 1.038 | 1.013-1.063 | 0.003 | 1.047 | 1.030-1.064 | 0.000 | 1.048 | 1.040-1.056 | 0.000 |
| **Gender** |  |  |  |  |  |  |  |  |  |  |  |  |
| Male | 1 |  |  | 1 |  |  | 1 |  |  | 1 |  |  |
| Female | 0.794 | 0.507-1.242 | 0.053 | 0.635 | 0.371-1.087 | 0.098 | 0.867 | 0.591-1.270 | 0.463 | 0.699 | 0.584-0.837 | 0.000 |
| **Marital Status** |  |  |  |  |  |  |  |  |  |  |  |  |
| Single | 1 |  |  | 1 |  |  | 1 |  |  | 1 |  |  |
| Married | 0.312 | 0.164-0.592 | 0.000 | 1.201 | 0.468-3.083 | 0.704 | 0.576 | 0.305-1.086 | 0.088 | 0.863 | 0.647-1.152 | 0.318 |
| Separated/divorced/widowed | 0.501 | 0.243-1.036 | 0.062 | 2.690 | 0.962-7.517 | 0.059 | 0.633 | 0.306-1.311 | 0.218 | 1.023 | 0.742-1.411 | 0.890 |
| **Ethnic group** |  |  |  |  |  |  |  |  |  |  |  |  |
| Non-Hispanic white | 1 |  |  | 1 |  |  | 1 |  |  | 1 |  |  |
| Hispanic white | 1.164 | 0.351-3.860 | 0.804 | 0.000 | . | . | 1.988 | 0.988-4.000 | 0.054 | 1.306 | 0.834-2.045 | 0.244 |
| Black | 1.392 | 0.414-4.682 | 0.593 | 0.402 | 0.053-3.054 | 0.379 | 1.901 | 0.912-3.961 | 0.086 | 1.612 | 1.064-2.441 | 0.024 |
| Asian | 2.424 | 1.054-5.572 | 0.037 | 0.920 | 0.323-2.620 | 0.876 | 0.239 | 0.033-1.744 | 0.158 | 1.311 | 0.901-1.909 | 0.157 |
| **Treatment** |  |  |  |  |  |  |  |  |  |  |  |  |
| No surgery or radiation | 1 |  |  | 1 |  |  | 1 |  |  | 1 |  |  |
| Surgery | 1.091 | 0.688-1.730 | 0.712 | 0.798 | 0.479-1.329 | 0.386 | 0.690 | 0.486-0.979 | 0.038 | 0.910 | 0.763-1.085 | 0.293 |
| Radiation | 0.702 | 0.372-1.325 | 0.275 | 0.435 | 0.216-0.876 | 0.020 | 0.361 | 0.050-2.623 | 0.314 | 1.122 | 0.788-1.598 | 0.523 |
| Radiation & Surgery | 0.609 | 0.245-1.516 | 0.287 | 0.150 | 0.036-0.632 | 0.010 | 0.736 | 0.331-1.638 | 0.453 | 1.279 | 0.849-1.927 | 0.238 |
| **B symptoms** |  |  |  |  |  |  |  |  |  |  |  |  |
| No | 1 |  |  | 1 |  |  | 1 |  |  | 1 |  |  |
| Yes | 2.079 | 0.970-4.458 | 0.060 | 1.784 | 0.931-3.418 | 0.081 | 2.325 | 1.495-3.614 | 0.000 | 1.473 | 1.210-1.794 | 0.000 |
| Unknown | 1.608 | 1.002-2.580 | 0.049 | 1.262 | 0.744-2.138 | 0.388 | 1.280 | 0.879-1.864 | 0.198 | 1.005 | 0.835-1.210 | 0.955 |
| **Extranodal involvement** |  |  |  |  |  |  |  |  |  |  |  |  |
| No | 1 |  |  | 1 |  |  | 1 |  |  | 1 |  |  |
| Yes | 0.567 | 0.358-0.897 | 0.015 | 0.711 | 0.441-1.147 | 0.163 | 0.601 | 0.307-1.177 | 0.138 | 0.906 | 0.710-1.157 | 0.431 |

Cancers diagnosed 1999-2004 and followed up to 12/31/2009. HR: hazard ratio.
